# Thoracoscopic sublobar resection for congenital bronchial atresia in adults: a report of three cases

**DOI:** 10.1186/s44215-023-00118-2

**Published:** 2023-11-14

**Authors:** Hironobu Samejima, Naoko Ose, Hideki Nagata, Soichiro Funaki, Yasushi Shintani

**Affiliations:** 1 Department of General Thoracic Surgery, National Hospital Organization Kinki-Chuo Chest Medical Center, 1180, Nagasone-cho, Kita-ku, Sakai, Osaka, 591-8555 Japan; 2 Department of General Thoracic Surgery, Osaka University Graduate School of Medicine, 2-2, Yamadaoka, Suita, Osaka, 565-0871 Japan

**Keywords:** Thoracoscopic surgery, Sublobar resection, Three-dimensional image reconstruction system, Bronchial malformation, Congenital bronchial atresia

## Abstract

**Background:**

Congenital bronchial atresia (CBA) is a rare bronchial abnormality. Adult patients with CBA generally do not show any symptoms. Although surgical resection is the first-line treatment, the indication for the surgical intervention is controversial, especially in mildly symptomatic or asymptomatic patients. Recently, thoracoscopic surgery is the commonly preferred approach to treat this condition.

**Case presentation:**

Case 1: A woman in her 20s suffered from repeating episodes of fever and cough due to left CBA with the interruption of B9 and B10. Uniportal thoracoscopic left lower lobe wedge resection was performed, following which the symptoms disappeared. Case 2: A woman in her 40s presented to the clinic with dyspnea on exertion, and the computed tomography image revealed the absence of B2. Multiportal thoracoscopic right S2 segmentectomy was performed on her, after which the symptoms of dyspnea improved. Case 3: A woman in her 40s visited the hospital with symptoms of intermittent cough, sputum, and slight fever for 7 months. The examinations identified the absence of B6a. Right S6 segmentectomy and S2 wedge resection were carried out by multiportal thoracoscopic approach. The patient is now asymptomatic.

**Conclusions:**

Based on our experiences in the three adult CBA surgery cases, the three-dimensional image reconstruction system is very useful for diagnosis and surgical decisions with regard to this disease. Even for CBA patients with subtle symptoms, early surgical treatments may be beneficial from the point of view of minimally invasive interventions, and sublobar resection will be sufficient for treatments and preservation of respiratory function.

## Background

Congenital bronchial atresia (CBA) is a type of bronchial malformation originating in the fetal period. CBA is associated with blind termination of the bronchus, resulting in mucoid impaction in the distal bronchi and emphysematous change in the obstructed lung parenchyma [1]. Although CBA in young children usually presents with fever or respiratory symptoms, most adult patients are diagnosed accidentally and are generally asymptomatic [2]. Surgical resection is the standard treatment, and the number of cases of thoracoscopic surgeries for treating this condition is on the rise [3].

Herein, we report three cases of adults suffering from CBA, who underwent thoracoscopic surgery after preoperative investigations using the three-dimensional (3D) image reconstruction system: SYNAPSE VINCENT® (FUJIFILM Corp.).

## Case presentation

### Case 1

**Fig. 1:**
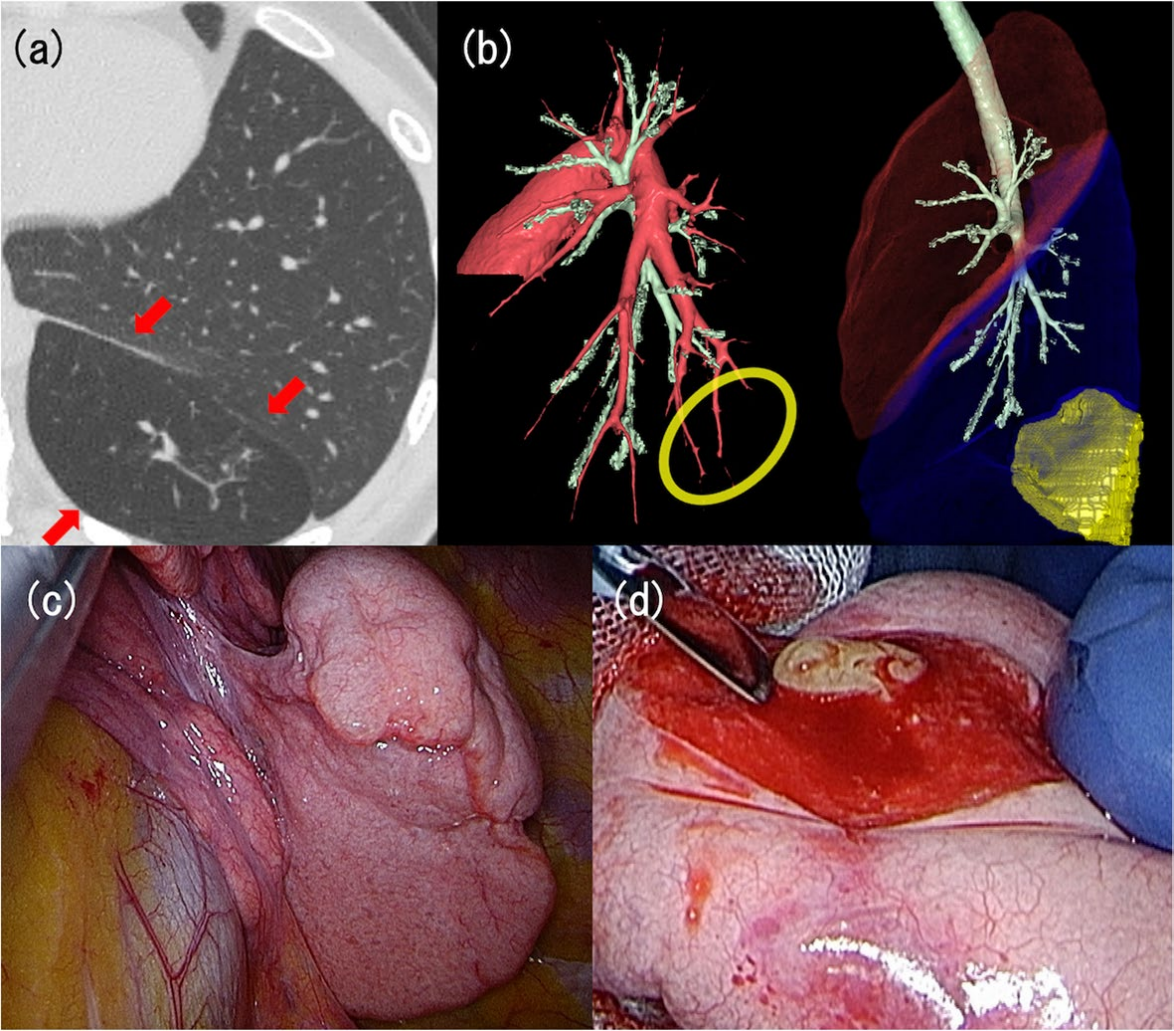
Chest computed tomography (CT) images and intraoperative findings of case 1. **a** Chest CT indicated the abnormal low-density area with an aberrant fissure in S9 and S10 in the left lung, as indicated by red arrows. **b** Two images obtained from three-dimensional reconstruction of chest CT findings: In the left image, red and green structures represent pulmonary arteries and bronchi, respectively. The yellow ellipse represents the abnormal region. In the right image, the red zone is the left upper lobe, and the blue zone is the left lower lobe. The green structures represent the bronchi. The yellow area indicates the lesion. Two images revealed no bronchi to the lesion. **c** The lesion attached to the normal lung by a peduncle. **d** Yellow mucus in the resected lung

A woman in her 20s visited the hospital with repeated fever and cough for 1 year. The patient had no significant medical history, and the blood tests identified no abnormalities. Chest contrast-enhanced computed tomography (CT) revealed a low-density area with an aberrant fissure in the peripheral region of S9 and S10 in the left lung, which also showed the presence of bronchial dilation and mucoid impaction (Fig. 1a). Moreover, the absence of bronchi in the area and interruption of B9 and B10 were indicated (Fig. 1b). The patient was diagnosed with left CBA.

The surgery was performed with uniportal approach. The lesion was attached to the normal lung by a peduncle (Fig. 1c). A wedge resection was carried out, and yellow mucus was observed in the resected lung (Fig. 1d). Pathological examination demonstrated mild emphysematous changes and bronchiectasis. The bacterial culture test conducted on the mucus was negative for infection. The postoperative course was uneventful. The patient returned to work and has remained asymptomatic after the surgery.

### Case 2

**Fig. 2:**
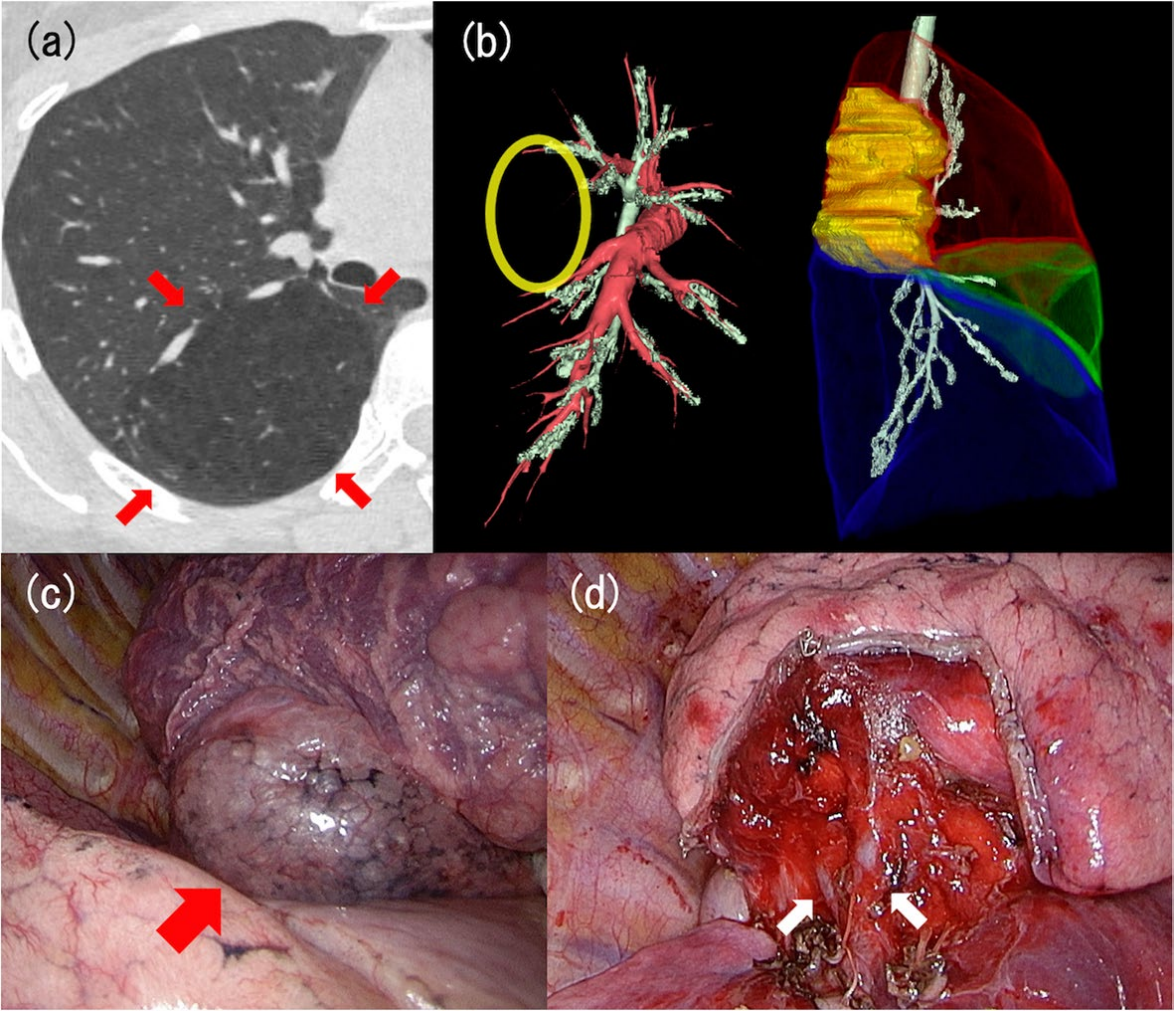
Chest computed tomography (CT) images and intraoperative findings of case 2. **a** Chest CT revealed a low-density area in right S2, as indicated by red arrows. **b** In the left image of the reconstructed chest CT, red structures represent pulmonary arteries, and green structures represent bronchi. The yellow ellipse represents the lesion. In the right image, red, green, and blue regions indicate the right upper, middle, and lower lobes, respectively. The green structures represent the bronchi. The yellow area is the low-density area. These findings demonstrated the absence of B2 and A2. **c** Emphysematous changes in S2 are indicated by a red arrow. **d** Intraoperative findings after resection of the lesion. V2a is indicated by the right white arrow, and V2c is indicated the left white arrow

A woman in her 40s presented with symptoms of dyspnea on exertion for 1 year. The medical history was insignificant. No abnormalities were detected in the blood tests. Chest contrast-enhanced CT revealed a low-density area in right S2 (Fig. 2a) and the absence of bronchi as well as pulmonary arteries in the S2 area (Fig. 2b). The pulmonary function was normal, and desaturation on effort was not observed. Her subjective dyspnea seemed to be due to the overinflated lesion of CBA.

Right S2 segmentectomy was performed by multiportal thoracoscopic approach. The CBA region, which showed emphysematous change, had clear boundaries with the normal lung (Fig. 2c). There were severe inflammatory changes in the interlobar area between upper and lower lobes. B2 and A2 were not observed, and only the V2b was dissected. The intersegmental plane was determined to facilitate removal of the entire emphysematous area (Fig. 2d). Histological examination demonstrated inflammatory changes, bronchiectasis, and mucoid impaction in the resected specimen. A bacterial culture test of the resected tissue was negative. The patient made favorable progress after the surgery, and her symptoms improved considerably.

### Case 3

**Fig. 3:**
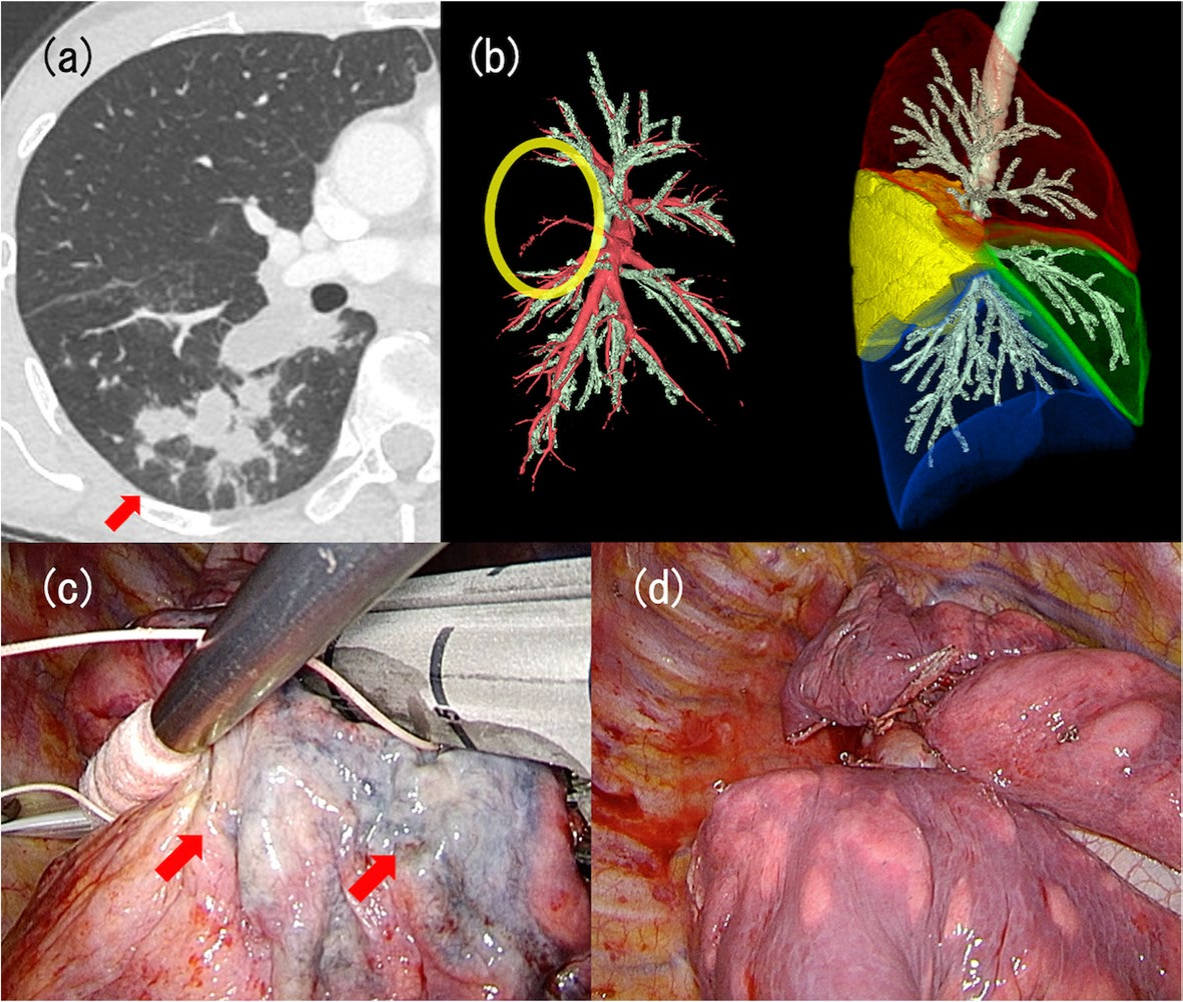
Chest computed tomography (CT) images and intraoperative findings of case 3. **a** Chest CT identified consolidation in right S6, as shown by a red arrow. **b** The two images were three-dimensional reconstructions of chest CT findings. In the left image, red and green structures indicate the pulmonary arteries and bronchi, respectively. The yellow ellipse indicates the area of the disease. In the right image, the right lung is separated into four zones by different colors. Red represents the upper lobe, green represents the middle lobe, blue represents the lower lobe, and yellow represents the lesion. The bronchi have been presented as the green branched structure. The image findings implied absence of B6a. **c** The color change of visceral pleura of S6 was observed, as indicated by red arrows. **d** The lesion was removed completely at the end of the surgery

A female amateur marathon runner in her 40s visited a nearby hospital, because she could not get enough exercise due to symptoms of intermittent cough, sputum, and slight fever for 7 months. The patient had a history of chronic cough 4 years earlier, which was diagnosed as cough variant asthma, and the symptoms had resolved following medical treatment. The blood tests showed slightly elevated levels of C-reactive protein at 0.44 mg/dL. Chest contrast-enhanced CT revealed consolidation mainly in right S6, and to a lesser extent in S2 (Fig. 3a) with the absence of B6a in the lesion (Fig. 3b).

Multiportal thoracoscopic right S6 segmentectomy and S2 wedge resection were performed. The thoracic cavity showed no adhesions, and only a slight pleural effusion was observed. There were no fissures between S2 and S6, whereas an aberrant fissure existed between S6 and basal segments. S6, predominantly the dorsal area, exhibited intensive emphysematous changes with insufficient deflation. S6 and partial area of S2b showed color change of the visceral pleura, which seemed to be caused by inflammation (Fig. 3c). The peribronchial tissues of B6 were particularly affected and stiff, probably because of inflammatory change. The intersegmental plane including the whole lesion was excised (Fig. 3d). Pathological examination indicated inflammatory change, bronchiectasis, and mucoid impaction. *Pseudomonas aeruginosa* was detected in the bacterial culture of the resected lung. There were no postoperative complications, and the symptoms disappeared, which has enabled her to enjoy exercise.

## Discussion and conclusions

The origin of CBA is thought to be induced by the focal insufficient blood supply of the bronchial artery prior to birth [1]. Although some reports have described that CBA appears after the 16th week of the fetal period after formation of bronchial branches is completed [4], some researchers state that CBA appears before 15 weeks of the fetal period, during the process of formation of bronchial branches [5]. According to past studies, ischemia partially inhibits development of the bronchus, resulting in bronchial atresia [1, 2]. However, the lung parenchyma and bronchi distal to the termination of the bronchus continue to develop normally because of alternate vascular supply. After birth, collateral ventilation causes trapping of air, leading to emphysematous changes in the distal lung parenchyma. Furthermore, bronchial atresia disrupts drainage of airway secretions in spite of the normal secretion of mucinous products, which leads to dilation of the distal bronchi and mucoid impaction [1]. CBA in the segmental or subsegmental bronchi is more common and accounts for > 80% of the cases; however, cases of lobar bronchial atresia are also routinely observed [5, 6]. The patients from our case studies suffered from segmental or subsegmental bronchial atresia.

Although surgical treatment is generally necessary for severely symptomatic or infected patients having CBA, there is no consensus about indications for treatment of mildly symptomatic or asymptomatic patients. Some reports suggest that surgery is warranted considering the risk of future infection and the possibility of compression of the normal lung caused by the overinflated lesion because of air trappings through the collateral ventilation routes [3, 7, 8]. However, there is another point of view that recommends conservative approach and observation [2, 4]. In the cases covered in this article, the symptoms were not serious but had a negative impact on the lives of the patients, and thoracoscopic sublobar resection brought about a remarkable improvement in the quality of their life.

With regard to surgical approach, thoracoscopic surgery without thoracotomy has recently been conducted whenever possible [3, 4, 7, 9]. While all the three cases described above were surgically treated using a thoracoscopic approach, inflammatory changes of the tissues were noticeable in cases 2 and 3. Inflammatory changes caused by infection make it difficult to visualize and identify the anatomical structure and to dissect the tissues, which occasionally requires a conversion to thoracotomy. Accordingly, even in mildly symptomatic or asymptomatic patients, early treatment could ensure a safer and less invasive surgery.

Moreover, in surgical decision, it is very important to preserve respiratory function because of the lesion being benign [3, 9]. For an atresia located in the segmental or more distal bronchus, sublobar resection is the recommended procedure. However, lobectomy is sometimes required due to the spread of infection and long-term compression of the normal lung adjacent to the overinflated lesion [1, 3, 7, 8]. As for our patients, the lesions were localized and had clear boundaries with the normal lung. Neither spread of infection to other segments nor compression of the normal lung was observed. Early sublobar resection should be considered even for patients with subtle symptoms because of the possibility that the volume of the resected lung might be larger owing to infection or overinflation of the lesion with passing time.

In conclusion, based on our experience and past studies, surgical treatments for patients with subtle symptoms would be recommended, because they are likely to recover completely by thoracoscopic sublobar resection [3, 7, 8]. Furthermore, surgery for asymptomatic patients may be a viable treatment option taking the future risk of this disease into consideration [3, 7, 8].

Regarding the diagnosis of this condition, chest CT is the most important examination, and the 3D image of chest CT obtained by employing the 3D image reconstruction system is helpful in the diagnosis and surgical decision [1, 3, 4]. When segmentectomy is conducted, it is essential to accurately understand the range of the lesion and the course covered by the bronchi and pulmonary arteries and veins. The 3D image reconstruction system proves to be very useful for this purpose. In our cases, the surgeries were carried out smoothly after referring to the reconstructed images. Moreover, emphysematous change associated with CBA causes boundaries between the normal lung and the affected area to be visualized clearly [3, 7]. The ranges of resection in the three cases could be decided easily based on intraoperative findings. Segmentectomy for this condition might not always require specialized means, such as the use of indocyanine green, for deciding the cut line of lung parenchyma.

We have reported three cases of CBA who underwent thoracoscopic sublobar resection following preoperative consideration using the 3D reconstruction system of chest CT image. Early surgery for patients with subtle symptoms enables performance of minimally invasive operations and preservation of respiratory function by reducing the volume of the resected lung.

## Data Availability

The datasets used and analyzed during the current study are available from the corresponding author on reasonable request.

## Abbreviations

CBA: Congenital bronchial atresia
CT: Computed tomography
3d: Three-dimensional
